# Bone imaging in prostate cancer: the evolving roles of nuclear medicine and radiology

**DOI:** 10.1007/s40336-016-0196-5

**Published:** 2016-07-20

**Authors:** Gary J. R. Cook, Gurdip Azad, Anwar R. Padhani

**Affiliations:** 1 Division of Imaging Sciences and Biomedical Engineering, Department of Cancer Imaging, Clinical PET Centre, St Thomas’ Hospital, Kings College London, London, SE1 7EH UK; 2 Paul Strickland Scanner Centre, Mount Vernon Cancer Centre, Rickmansworth Road, Northwood, Middlesex HA6 2RN UK

**Keywords:** Prostate cancer, Bone metastases, Bone scan, Positron emission tomography, Single photon emission computed tomography, Whole body magnetic resonance imaging

## Abstract

The bone scan continues to be recommended for both the staging and therapy response assessment of skeletal metastases from prostate cancer. However, it is widely recognised that bone scans have limited sensitivity for disease detection and is both insensitive and non-specific for determining treatment response, at an early enough time point to be clinically useful. We, therefore, review the evolving roles of nuclear medicine and radiology for this application. We have reviewed the published literature reporting recent developments in imaging bone metastases in prostate cancer, and provide a balanced synopsis of the state of the art. The development of single-photon emission computed tomography combined with computed tomography has improved detection sensitivity and specificity but has not yet been shown to lead to improvements in monitoring therapy. A number of bone-specific and tumour-specific tracers for positron emission tomography/computed tomography (PET/CT) are now available for advanced prostate cancer that show promise in both clinical settings. At the same time, the development of whole-body magnetic resonance imaging (WB-MRI) that incorporates diffusion-weighted imaging also offers significant improvements for detection and therapy response assessment. There are emerging data showing comparative SPECT/CT, PET/CT, and WB-MRI test performance for disease detection, but no compelling data on the usefulness of these technologies in response assessment have yet emerged.

## Introduction

The skeleton is the second commonest site for metastases from prostate cancer after lymph nodes, and there is an incidence of 65–75 % of skeletal involvement in patients with advanced disease [1]. Skeletal metastases are associated with significant morbidity and skeletal related events; however, effective palliation strategies are available resulting in improvements in overall survival (currently 12–53 months), longer than with most other types of cancer that have metastasised to bone [2]. Management of prostatic bone metastases has a significant impact on health care resources [3].

It is recognised that conventional imaging, including radiographs, computed tomography (CT) and bone scintigraphy (BS), is relatively insensitive and/or non-specific for the diagnosis of skeletal metastases. In addition, commonly used response assessment methodologies, such as response evaluation criteria in solid tumours (RECIST 1.1) [4], do not adequately cater for bone disease response, especially in osteoblastic disease which is the commonest manifestation in metastatic prostate cancer. Improvements made in detection with modern hybrid and functional methods, including positron emission tomography (PET)/CT, single-photon emission computed tomography (SPECT)/CT, whole-body magnetic resonance imaging (WB-MRI) and PET/MRI, and the relative merits of these methods will be reviewed in this study. However, the ability to provide a timely and accurate assessment of treatment response, so as to maximise the use of successful treatments and minimise exposure to non-effective treatments (and their toxicities), is less well studied by modern imaging techniques. Nevertheless, there are accumulating data that functional or hybrid imaging is likely to offer superior efficacy in therapy assessment, and these aspects will also be discussed.

## Pathophysiology of bone metastases

Spread of prostate cancer to bone is via the haematogenous route, whereby cancer cells initially settle in the bone marrow where they are able to grow in accordance with the “seed and soil” hypothesis originally proposed by Paget [5]. In bone metastases from prostate cancer, there is predominant upregulation of osteoblastic activity leading to the formation of mineralised woven bone, causing the characteristic osteosclerotic appearance on radiographs and CT, but it is recognised that osteoclasts also play an important part in the pathophysiology of the metastatic growth process [6]. A key mechanism of control is tumour cell influence over osteoblast/osteoclast activity, through the expression of cytokines, including the receptor activator of the nuclear factor-κB ligand (RANKL), an activator of osteoclast differentiation. Increased osteoblastic, osteoclastic, and tumour cell activity are not only therapeutic targets but also represent potential targets for imaging. Other altered imagable bone targets include trabecular bone density, neoangiogenesis, bone marrow fat, water and iron content and tumour related macrophages.

### Tumour cells

Abnormal tumour metabolism may be depicted by a variety of PET tracers. For example, increased membrane synthesis that occurs in proliferating tumours is associated with increased accumulation of choline (e.g. ^11^C or ^18^F-choline tracers) [7], enhanced fatty acid metabolism (e.g. ^11^C-acetate) [8] or amino acid transport (e.g. anti-1-amino-3-[18F]fluorocyclobutane-1-carboxylic acid, FACBC or fluciclovine) [9]. Prostate cancer cells typically do not show significant ^18^F-fluorodeoxyglucose metabolism unless dedifferentiated and castrate-resistant [10]. Tracers that image specific aspects of prostate cancer cellular biology include 16beta-^18^F-fluoro-5alpha-dihydrotestosterone (^18^F-FDHT) for androgen receptor targeting [11] and prostate-specific membrane antigen labelled with ^68^Gallium (^68^Ga-PSMA) [12, 13]. Tumour cell infiltration within the bone marrow can also be detected on morphological T1/T2-weighted MRI sequences but can also be detected by diffusion-weighted (DW) sequences; the latter is sensitive to the increased impediment of water molecule motion in hypercellular tissues [14]. In addition, the displacement of normal bone marrow fat by tumour cells and matrix mineralisation can also be detected on gradient-echo imaging sequences which enable the separate imaging of bone marrow water and fat [15, 16].

### Osteoblastic activity

Increased osteoblastic activity leads to an osteosclerotic appearance on radiographs and CT and causes increased accumulation of bone-specific tracers, such as ^99m^Tc-methylene diphosphonate (^99m^Tc-MDP) (SPECT) or ^18^F-fluoride (PET). The high osteoblastic activity in metastases from prostate cancer compared to other tumours means that these methods have traditionally shown good sensitivity. However, an increase in osteoblastic activity frequently occurs in bone metastases responding to treatment and bone-specific tracers may, therefore, be unable to differentiate an increase or new uptake due to osteoblastic healing (flare phenomenon) from an increase/new activity due to progressive disease [17]. Another limitation of using an indirect osteoblastic detection process for detecting metastatic disease includes missing or underestimating the volume of disease that does not incite an osteoblastic reaction.

### Osteoclastic activity

Osteoclasts lead to bone destruction and osteolytic lesions, and whilst this is a less common appearance in prostate cancer, it may be observed on radiographs and CT. The presence of marked osteolysis should prompt histologic reevaluation, because it can indicate the emergence of aggressive prostate cancer variants, which are increasingly seen in the later stages of the metastatic process after several rounds of treatments. Osteoclasts express high levels of the integrin α_v_β_3_ that facilitates adherence of osteoclasts to the endosteal bone surface, promoting resorption. PET and SPECT tracers that were originally designed to target α_v_β_3_ integrin in tumour-related angiogenesis with the arginine–glycine–aspartic acid (RGD) sequence have shown some utility in targeting osteoclastic activity in bone metastases in animal models and humans [18–20].

## Staging

### Bone scans

Due to the low incidence of skeletal metastases in patients with a new diagnosis of low-risk prostate cancer (e.g. PSA < 10 ng/ml, Gleason score < 8, no bone pain), a staging bone scan is not recommended [21], and some have refined risk factors, e.g. PSA < 20 ng/ml, stage < T4 and Gleason < 8 [22]. In those with an increased risk of bone metastases, the bone scan with tracers such as ^99m^Tc-MDP has been the commonest method for detecting skeletal involvement [23]. However, it is recognised that it may not detect small bone marrow-based metastases that have not caused a large enough osteoblastic response to be identifiable. For disease limited to the bone marrow, WB-MRI or PET imaging may be more sensitive [24]. Another perceived weakness of bone scans is a lack of specificity as non-malignant skeletal disease may also cause focal uptake of bone-specific tracers, often requiring further correlative morphological imaging (e.g. radiographs, MRI and CT). With modern hybrid imaging, improved characterisation of the causes of bone scan uptake has largely been addressed, such that morphological appearances can help correctly attribute non-malignant scintigraphic uptake, thus improving specificity and reducing the number of equivocal interpretations. This has been shown with ^99m^Tc-MDP SPECT/CT in patients with prostate cancer where compared with planar imaging, the number of equivocal lesions dropped from 61 to 8 % with the addition of SPECT/CT [25].

Whilst the flare phenomenon may cause false positives when assessing treatment response (see below), it may be useful in diagnosing bone metastases in new patients who are started on hormone therapy. A second bone scan 6 weeks after commencing hormones may show either new, previously occult lesions, or an increase in uptake in previous equivocal lesions, thereby improving both sensitivity and specificity in detection of skeletal disease [26].

### ^18^F-fluoride PET

^18^F-fluoride was introduced as a bone-specific tracer more than 50 years ago [27] and has uptake mechanisms similar to diphosphonates (e.g. ^99m^Tc-MDP) that rely on blood flow and local osteoblastic activity [28]. However, it was not until the improvement in PET scanners in more recent years, that it was possible to take advantage of the superior imaging characteristics compared to bone scintigraphy. ^18^F-fluoride shows rapid skeletal uptake and background soft tissue clearance allowing high-quality skeletal imaging as soon as 1 h after injection. These characteristics, combined with superior spatial resolution and tomographic acquisitions as a routine, improve diagnostic accuracy in patients with prostate cancer compared with bone scan [29]. A prospective study of 44 patients with high-risk prostate cancer showed superiority of ^18^F-fluoride PET/CT over ^18^F-fluoride PET, bone scan augmented with SPECT and planar bone scan alone, the respective sensitivities, specificities, positive and negative predictive values being reported as PET/CT: PET: BS + SPECT and BS: 100, 100, 100, 100 vs 100, 62, 74, 100 vs 92, 82, 86, 90 vs 70,57, 64 and 55 % [28]. In a report from the National Oncologic PET Registry in the USA that assessed the effects of ^18^F-fluoride PET/CT in prostate cancer, bone metastasis was confirmed in 14 % at initial staging and 29 % in those with suspected first osseous metastasis [30]. The post-imaging plan was revised to treatment in 77 % and 52 % in these respective groups.

### ^18^F-FDG PET

Despite the widespread utility in most cancers, ^18^F-FDG PET appears to have limited sensitivity in detecting skeletal metastases from prostate cancer. Compared with bone scans, ^18^F-FDG PET detected 64 out of 100 bone scan positive lesions in patients with a new diagnosis of prostate cancer and only 4 out of 131 in patients receiving hormone deprivation therapy, in whom PSA levels ranged from 499 to 4786 ng/ml [31]. In another study of patients with hormone-resistant disease, only 18 % of bone scan lesions showed accumulation of ^18^F-FDG and the authors concluded that lesions show a low glycolytic rate and that other metabolic mechanisms may be more dominant in prostate cancer [32]. It has also been reported that in castrate resistant prostate cancer patients with ^18^F-FDG avid metastases, the number and extent of ^18^F-FDG avid disease correlates with survival [33].

### ^18^F/^11^C-choline PET

Both ^18^F-choline and ^11^C-choline probably show similar diagnostic accuracy. Detection of bone metastases in 140 patients has been reported with ^11^C-choline. Uptake was seen in both osteoblastic (*n* = 97) and osteolytic lesions (*n* = 43) but with significantly higher SUVmax in osteolytic disease [34]. Another study showed that densely sclerotic lesions (CT Hounsfield Units > 825) did not show ^18^F-choline uptake. It was noted that nearly all of these patients had received hormone therapy and the lack of activity was interpreted as being due to a treatment effect resulting in non-viable bone metastases [35].

Most studies have shown a higher diagnostic accuracy for choline PET/CT compared with bone scans for initial staging or specifically in the spine [36–38]. One study reported a lower sensitivity but higher specificity and a fewer equivocal lesions [39]. In comparison with ^18^F-fluoride PET/CT, ^18^F-choline has been reported to show a non-statistically significant lower sensitivity (74 vs 81 %), a higher specificity (99 vs 93 %, *p* = 0.01) and no difference in overall accuracy (85 vs 86 %) [40]. Two patients had bone marrow lesions detected with ^18^F-choline with a change in management, and although some patients had more lesions detected with ^18^F-fluoride, management was not changed in these. A similar comparison between ^18^F-choline and ^18^F-fluoride by the same group found no statistically significant differences overall in a group of 42 patients but a better specificity for ^18^F-choline in a subgroup of patients with suspected recurrence (96 vs 91 %, *p* = 0.03) [41]. As a tumour-specific tracer, choline is able to detect both bone and soft tissue metastases.

### Other PET tracers

In patients with biochemical recurrence of prostate cancer, ^11^C-acetate has shown high concordance with bone scans with a sensitivity of 90 % and specificity of 96 % [42]. In an interesting comparison of ^18^F-FDHT and ^18^F-FDG with CT in patients with castrate resistant prostate cancer, an inverse correlation was reported between uptake of both tracers and CT lesion density. The number of skeletal metastases on CT and both PET methods predicted survival. Uptake of ^18^F-FDHT, but not ^18^F-FDG, showed an inverse correlation with survival [33].

There is a strong interest in assessing the role of prostate specific membrane antigen (PSMA) targeting tracers for SPECT and PET imaging given that most prostate cancer cells highly overexpress this target. In patients with biochemical recurrence of prostate cancer, ^68^Ga-PSMA has shown higher sensitivity in bone and soft tissue disease with greater lesion conspicuity, particularly in bone [12, 43]. An early description of an ^18^F-labelled PSMA tracer analogue suggests superiority over bone scan and ^18^F-fluoride PET [13], but a formal comparison has not yet been made in a substantive study. Early data also suggest that ^68^Ga-PSMA has a greater sensitivity for disease detection than choline-PET/CT in patients with biochemical recurrence, especially at low PSA values [12, 44]. The ability of PSMA PET/CT to evaluate therapy response has not been systematically evaluated.

### Whole-body MRI (WB-MRI)

Several meta-analyses have shown improved bone and soft tissue disease detection performance of WB-MRI comparable with ^18^F-FDG PET/CT, both being significantly more accurate than bone scans and CT, in the majority of solid cancers, on a per patient and per lesion basis [45–48]. The improved test performance of WB-MRI applies to skeletal assessments in advanced prostate cancer specifically, when choline PET/CT is used as the comparator technique. Shen et al. conducted a meta-analysis of 27 studies in advanced prostate cancer and showed that MRI was superior to choline PET/CT and BS on a per-patient basis [46]. On a per-patient basis, the pooled sensitivities for bone disease using choline PET/CT, WB-MRI and BS were 91 % (95 % CI 83–96), 97 % (95 % CI 91–99) and 79 % (95 % CI 73–83), respectively. The pooled specificities for bone metastases detection using choline PET/CT, WB-MRI and BS were 99 % (95 % CI 93–100), 95 % (95 % CI 90–97) and 82 % (95 % CI 78–85), respectively. On a per-lesion analysis, choline PET/CT had a higher diagnostic odds ratio which exceeded both BS and bone SPECT for detecting bone metastases. Recent studies indicate that diffusion sequences contribute strongly to the enhanced diagnostic capability of WB-MRI. For example, Liu et al. [47] evaluated 32 studies with 1507 patients and showed a pooled sensitivity, specificity and the area under the curve for DWI of 95 % (95 % CI 90–97), 92 % (95 % CI 88–95) and 0.98 on a per-patient basis and 91 % (95 % CI 87–94), 94 % (95 % CI 90–96) and 0.97 on a per-lesion basis.

## Response assessment

### Bone scans

**Fig. 1:**
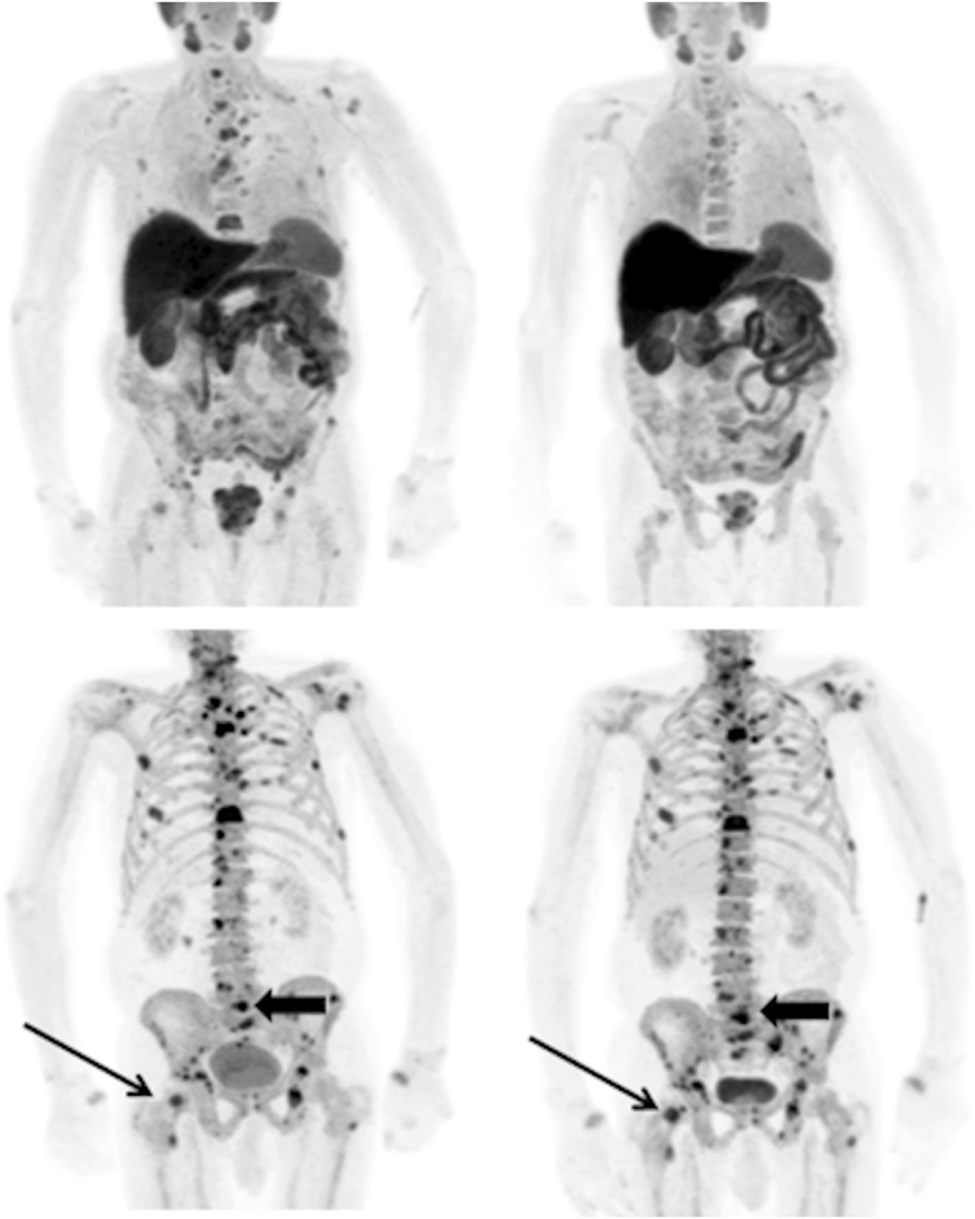
A patient with metastatic prostate cancer undergoing treatment with docetaxel chemotherapy. Top row ^11^C-choline PET maximum intensity projection images at baseline (*left*) and 8 weeks (*right*) and bottom row corresponding ^18^F-fluoride PET images. The higher contrast between metastases and the normal skeleton on the ^18^F-fluoride scans compared to the ^11^C-choline scans allows easier detection of disease. However, whilst there is a clear metabolic response in the bone metastases on the ^11^C-choline scans, there is a similar distribution and intensity of most lesions on the ^18^F-fluoride scans and some lesions show an increase in activity (arrows). This is likely to be due to a flare response at 8 weeks on the ^18^F-fluoride PET scans limiting the sensitivity and specificity in response prediction at early time points with this tracer as changes in osteoblastic activity lag behind changes in tumour metabolism

**Fig. 2:**
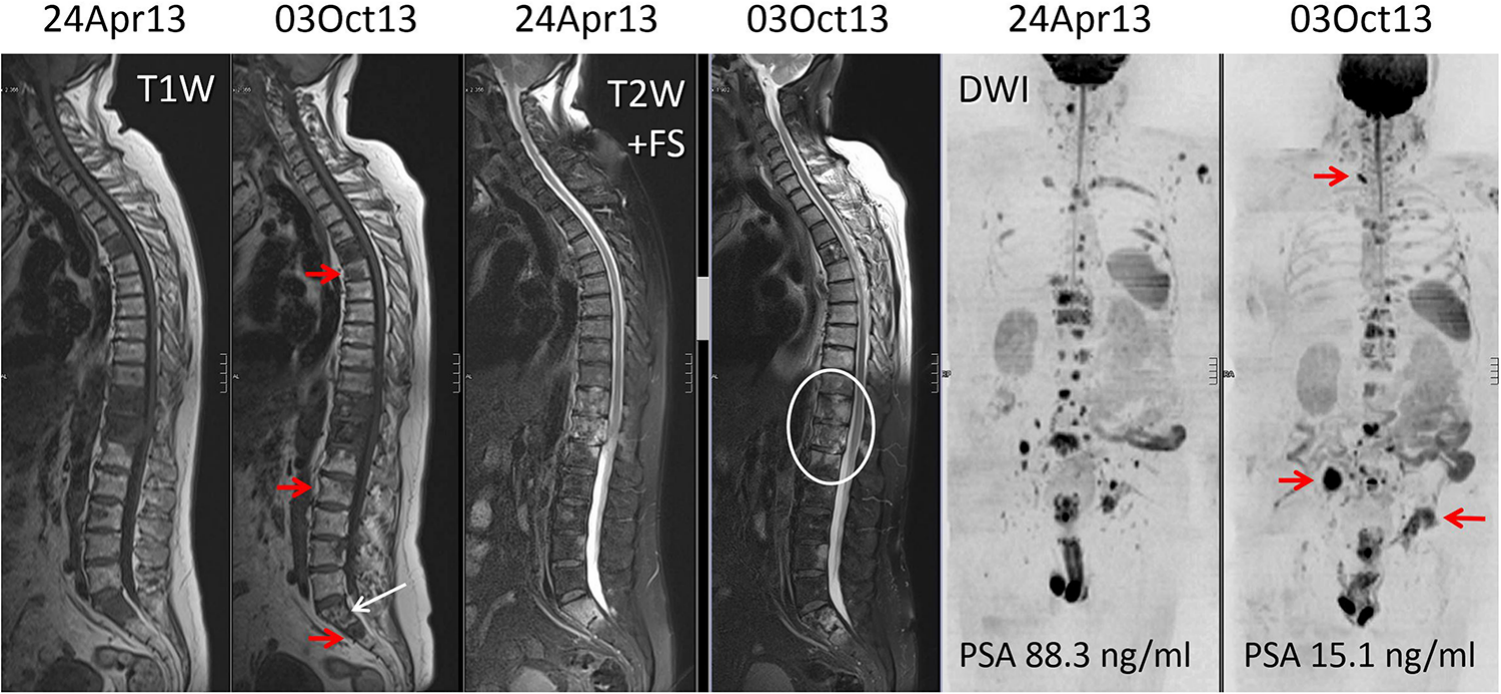
A 64 year old man with metastatic castrate resistant prostate cancer. WB-MRI assessments before and after five cycles of abiraterone therapy. The panel pairs are morphologic T1-weighted (*left*) and fat-suppressed T2-weighted (*middle*) sequences, and high *b* value (*b* 900 s/mm^2^) diffusion-weighted images (*right*) displayed as inverted MIP images. There is a discordant response to therapy documented on the imaging despite reductions in serum PSA levels. The *white arrows* and *ring* show decrease in tumour in the sacrum with return of normal marrow fat and relief of the spinal cord compression on the fat-suppressed T2-weighted sequence. However, the *red arrows* show disease progression in the spine, right iliac bone and *left* acetabulum

Bone metastases are notoriously difficult to assess for treatment response. RECIST1.1 does not fully cater for response assessment in bone, particularly with sclerotic metastases, and therefore, the Prostate Cancer Working Clinical Trials Group (PCWG) devised a framework specifically for prostate cancer response assessment with a focus on clinical trials [48, 49]. The criteria only allow for progressive disease, i.e. for patients in whom therapy needs to be changed or taken off trial. Bone scintigraphy is considered the standard imaging test and two new lesions are required on follow-up scans to determine progression as long as two new additional lesions are subsequently seen at least 6 weeks later. This is to control for false positives caused by a flare. PCWG recognises that there may be heterogeneity in response between metastases and also recognises alternative imaging methods, including ^18^F-fluoride PET, ^18^F-FDG PET, ^18^F-choline PET and bone marrow/body MRI, but that these should be considered as new biomarkers and are subject to independent validation (see Figs. 1, 2).

### ^18^F-fluoride PET

It is likely that the flare phenomenon will hamper response assessment in bone metastases given the similar mode of uptake to bone scan agents [50]. However, two small studies have shown changes in ^18^F-fluoride activity following specific treatments, including ^223^Ra-chloride [51] and dasatinib [52]. Total tumour burden measured on baseline ^18^F-fluoride PET has also been found to be a predictor of survival and skeletal related events in patients subsequently treated with ^223^Ra-chloride [53]. In an interesting case report, a corresponding appearance of progressive disease was seen on both ^18^F-fluoride and ^68^Ga-PSMA PET/CT following six cycles of ^223^Ra-chloride therapy [54]. In a National Oncologic PET Registry study on the effects of ^18^F-fluoride PET/CT on monitoring of systemic cancer therapy (68 % of patients with prostate cancer, 1940 scans), a change in management was recorded in 42 % of patients [55].

### ^18^F-FDG PET

Whilst metastases from prostate cancer are characteristically not very ^18^F-FDG avid, dedifferentiated disease in castrate resistant prostate cancer (CRPC) may show increased glycolytic activity. It has been reported that changes in ^18^F-FDG uptake correctly categorised 20/22 patients being treated with an antimicrotubule agent at 4 weeks compared with PCWG PSA criteria [56]. It was found that a 33 % increase in SUVmax or the appearance of a new lesion optimally divided progressors from non-progressors.

### ^18^F/^11^C-choline PET

There is a surprising lack of data on the use of PET choline tracers in assessing treatment response in bone metastases given the relatively high sensitivity for detection of disease. This use is supported by preclinical data showing reductions in ^11^C-choline activity in PC3 xenografts following treatment with docetaxel as soon as 1 week after commencing therapy [57]. Initial data in man are slightly conflicting. In a recent study, ^11^C-choline PET/CT changes between baseline and after completing treatment with docetaxel were found useful in identifying progression despite an apparent PSA response in a subset of patients [58]. A relationship between changes on ^18^F-choline PET activity and circulating cell-free DNA has been reported in a small series of eight patients, the authors concluding that the inter-related measures are potential markers of therapeutic response in CRPC [59]. In determining response to enzalutamide, one study showed that only baseline SUVmax of ^18^F-choline PET was a predictor of PFS and OS [60], whilst another reported that ^18^F-choline PET/CT does not add more information on OS than PSA alone [61]. In contrast, early ^18^F-choline PET/CT (3 and 6 weeks) has been reported to be able to predict clinical outcome in CRPC following abiraterone therapy beyond PSA response [62]. The potential value of ^18^F-choline has been described in two patients receiving ^223^Ra-chloride therapy with a reduction in lesion SUVs as well as in tumour burden parameters in a responding patient and heterogeneous response in a second patient [63].

### WB-MRI

Morphologic sequences are key for the confident detection of new metastases until the time when diffuse disease occurs after which the detection of disease reactivation becomes problematic. Morphologic criteria for bone disease progression and response are well described in the literature [64]. Specific clinical data on the use of morphological MRI in the routine assessment of metastatic bone disease response in advanced prostate cancer are lacking [65]. There are a number of problems encountered when using morphologic MRI to assess response, which includes arrested resolution of abnormalities despite effective therapy (the ‘residual scar’ phenomenon). Another limitation is the problem of evaluating disease activity on a scarred background of previously treated disease. A “T1 W image pseudoprogression—flare phenomenon” can also occur because of intense bone marrow oedema following tumour cell kill and inflammation, but its frequency is undocumented.

Both preclinical and small-scale clinical studies indicate that diffusion MRI can be useful for the assessment of therapy response in malignant bone marrow disease in prostate cancer. Preclinical mouse model studies of osseous prostate cancers have shown increases in diffusivity values with therapeutic success [66–68]. However, there have been a few systematic studies in prostate cancer patients with bone disease in the response assessment setting [69, 70]. The study of Reischauer et al. found that mean diffusivity of lesions increased significantly after hormonal therapy in keeping with successful responses gauged by PSA declines [69]. Interestingly, there was also noticeable spatial heterogeneity within individual metastases, with the centre of the lesions having greater increases in water diffusivity as well as variations between metastases in individual patients. Similar findings in bone disease have been described for multiple myeloma, myeloproliferative diseases, breast cancers and primary bone tumours with a variety of treatments, indicating that bone tumour diffusivity increases with successful treatments, and is a generic finding [71–74].

## Conclusion and future directions

There is no doubt that modern imaging methods, including PET/CT with bone-specific and tumour-specific tracers and WB-MRI with DWI, can improve both detection and therapy response assessment of patients with skeletal metastases from prostate cancer. However, it is not yet proved that earlier and more accurate detection of tumour presence and load will have positive therapy implications. It is also not clear that better categorisation of bone metastases response to therapy will have positive benefits. Nevertheless, there are strong indications that more accurate assessments of therapy response (including heterogeneity of response) could further aid the rational development of targeted therapies.

To address these questions, there is a strong need to standardise the evaluation, interpretation and reporting of PET/CT and WB-MRI technologies. By improving the evaluation of metastatic disease presence, load and response, a more complete characterisation of the metastatic state can be obtained, not only at the start of treatment, but also over time as the disease evolves. Whole-body PET/CT and WB-MRI technologies would also enable the evaluation of the benefits of continuing therapy, when there are signs that the disease is progressing. Neither PET/CT nor WB-MRI is at the point where they can support regulatory approvals of new therapeutic approaches in prostate cancer. Thus, we recommend that choline and PSMA PET/CT and WB-MRI are now evaluated in clinical trials to assess their impact on the clinical management of advanced prostate cancer patients.
